# Platelet-activating factor modulates apoptotic gene expression in developing sea urchin embryos

**DOI:** 10.3389/fcell.2026.1728206

**Published:** 2026-02-03

**Authors:** Shohom Saha, Aaron Cho, Caroline Smith, William E. Roudebush, Renee J. Chosed

**Affiliations:** Department of Biomedical Sciences, University of South Carolina School of Medicine Greenville, Greenville, SC, United States

**Keywords:** apoptosis, caspase, platelet-activating factor, sea urchins, signaling / signaling pathways

## Abstract

Examining gene expression patterns during early embryonic development is essential for understanding genetic disorders and fertility. The accessibility, cost-effectiveness, and reproducibility of sea urchin embryos have offered an effective model for studying how gene expression is regulated in early embryogenesis under the influence of specific growth factors. However, regulation of apoptotic gene expression by growth factors, such as Platelet-Activating Factor (PAF), during early embryogenesis remains unexamined. This study investigated the role of PAF during the first 4 hours post-fertilization in sea urchin (*Lytechinus variegatus)* embryos. Using quantitative real-time polymerase chain reaction (qRT-PCR), we analyzed expression patterns of apoptosis-promoting genes (*CASP3*, *CASP7* and *CASP8*) and apoptosis-inhibiting genes (*BCL2A1*, *NFKBIA*, *NFKBIZ*) in PAF-treated and untreated embryos collected at 20-min intervals for up to 240 min of development post-fertilization. This study revealed that PAF increases the expression of both pro-apoptotic caspase genes across all time points and anti-apoptotic genes in a biphasic manner. Our results demonstrate complex regulatory mechanisms that maintain the function of apoptotic machinery while preventing excessive cell death during critical early developmental stages.

## Introduction

The study of gene expression patterns during early embryonic development is essential for understanding developmental disorders and can also be used to understand developmental regulatory processes. Sea urchins can provide valuable insights into embryonic development due to their rapid maturation through multiple stages, transparent embryos, and conserved genetic pathways that are similar to those in human embryonic development (McClay, 2011). Sea urchin embryos are ideal for examining how gene expression is regulated in early embryogenesis under the influence of growth and environmental factors, particularly concerning genes involved in apoptosis. Their aquatic development allows for precise experimental manipulation of the culture environment, enabling controlled exposure to growth factors and other signaling molecules throughout embryogenesis (McClay, 2011; Adonin et al., 2021).

Apoptosis plays an important role in embryonic development by removing defective cells. Researchers have found that sea urchin apoptotic functions closely resemble human apoptotic functions, particularly in the Caspase pathway, and evolutionary conservation of apoptotic genes between sea urchins and humans supports the extension of results in sea urchins to human development (Adonin et al., 2021; Thurber and Epel, 2007; Galasso et al., 2019; Robertson et al., 2006). In addition, there are several identified common apoptotic genes conserved from humans and urchins, including *CASPASE*, *BCL-2*, and *NFKB* gene families (Galasso et al., 2019).

The importance of apoptosis extends beyond tissue sculpting to developmental resilience. Ramos-Ibeas et al. reported that apoptosis serves as a protective mechanism in bovine embryos, maintaining genomic stability and ensuring viable cell counts during *in vitro* fertilization (Ramos-Ibeas et al., 2020). Environmental stressors such as temperature fluctuations and chemical exposures can trigger apoptotic responses in sea urchin embryos, serving as a defense mechanism to remove irreversibly damaged cells (Vega and Epel, 2004). Thurber and Epel demonstrated that apoptotic timing during sea urchin development can be influenced by environmental factors, with apoptosis occurring primarily during later stages, such as gastrulation, to facilitate environmental adaptation (Thurber and Epel, 2007).

Beyond these genetic and environmental regulators, additional signaling molecules also influence apoptotic processes during embryogenesis. Although thorough studies exist on embryo development, critical gaps remain in understanding how the growth hormone Platelet Activating Factor (PAF: 1-alkyl, 2-acetyl phosphatidylcholine) affects gene expression and apoptotic function during early development. PAF is a phospholipid growth factor that regulates apoptosis, cellular signaling, and embryo viability across species (O'Neill, 2005; O’Neill, 2008). Berdyshev et al. established sea urchins as a model system for PAF research in embryology, demonstrating that PAF enhances sperm motility and fertilization capacity at specific concentrations (Berdyshev et al., 1995). Roudebush et al. identified PAF as a growth hormone influencing cellular processes such as apoptosis, embryo viability, and health across mammals (Roudebush et al., 2003). For sea urchins, this role remains unclear, especially within initial developmental stages. Lal et al. offered only a snapshot of intermediate developmental stages, while Adonin et al. emphasized the importance of investigating early development but did not analyze PAF-mediated regulation of apoptotic genes (Lal et al., 2017; Adonin et al., 2021).

Lal et al. demonstrated that PAF reduces Caspase-3 activity in sea urchins, crucial in apoptotic pathways, as excessive apoptosis can impair development by unnecessarily removing cells, while insufficient apoptosis may allow anomalies to persist (Lal et al., 2017). Roudebush et al. found that PAF exposure in rabbit and mouse embryos improved survival rates, embryo health, and accelerated developmental milestones (Roudebush et al., 2003). Lal et al. also observed that PAF-treated sea urchin embryos reached gastrula faster, highlighting PAF’s evolutionary importance as a regulatory hormone (Lal et al., 2017). However, overexpression might bypass essential quality control mechanisms. Yet, excessive suppression of apoptosis may allow survival of aneuploid or mutated cells, potentially leading to developmental abnormalities.

This study aims to address the major knowledge gap by investigating PAF’s role in early embryonic development through time-course experiments, specifically exploring how PAF affects apoptotic gene expression during early sea urchin development (O’Neill, 2005; O’Neill, 2008). Understanding how PAF influences apoptotic gene expression will contribute to a deeper understanding of developmental biology with potential implications for human embryology and infertility treatment. Yang et al. (2021) demonstrated that depletion of aneuploid cells through apoptosis is critical for human embryo development, while Nguyen et al. (2025) found that apoptotic gene expression patterns in human embryo culture media correlate with maternal age and embryo quality. PAF’s role in regulating apoptosis during early development may therefore be relevant to improving embryo selection and viability in human *in vitro* fertilization, where optimal apoptotic regulation could enhance developmental competence while maintaining quality control mechanisms (Roudebush et al., 2003).

## Methods

### Sea urchin gamete collection

Individual sea urchins (*Lytechinus variegatus)* were injected with 1 mL of 0.5 M potassium chloride into each side of the mouth orifice to trigger gamete shedding. Injections would continue until all sea urchins in the shipment batch of 12 were injected or two pairs of healthy males and females were found. Healthy egg and sperm batches were pooled accordingly. To ensure the formation of viable embryos and preclude anomalous factors that could affect our results, only healthy eggs, characterized by a vibrant, translucent orange color and healthy sperm with proper flagella, were used for our *in-vitro* fertilization studies. A sample was classified as healthy if approximately 80% of the eggs or sperm in the sample met the criteria when observed under a Nikon SMZ stereo microscope.

### Sea urchin embryo treatment and culture

**FIGURE 1:**
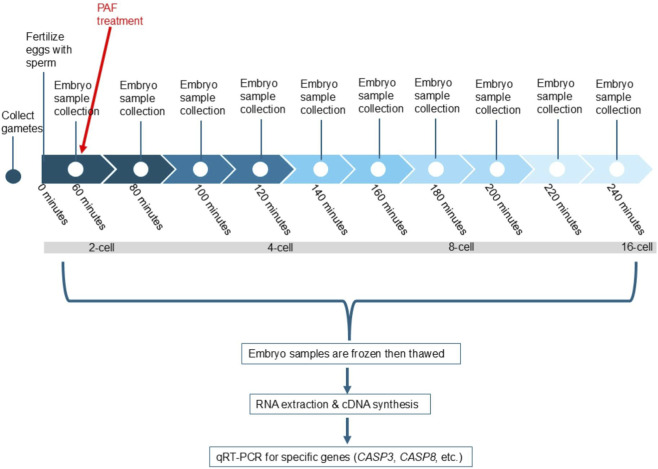
Timeline summary of methods performed in this study.

Approximately 500 eggs were inseminated with 2 µL of diluted spermatozoa in 100 µL filtered artificial seawater microdrops under oil in 60 mm Petri dishes. Samples were incubated for 1 h of fertilization to allow embryos to reach the two-cell stage (Figure 1), and then the experimental group was treated with 10^−7^ M PAF, followed by the collection of samples every 20 min for both control and experimental groups. Embryo development was terminated by collecting the sample via pipette and then storing it in microcentrifuge tubes before freezing at −80 °C. Each PAF-treated and control embryo culture had three biological replicates (from samples collected in summer 2024 and 2025) and two technical replicates to eliminate error. PAF (Cayman Chemical, USA) was used at a concentration of 10^−7^ M as this is similar to the biologically effective level to enhance embryo viability as reported by Nishi et al. (1995).

### RNA analysis using quantitative real-time polymerase chain reaction (qRT-PCR)

Total RNA was extracted from sea urchin embryos using the Zymo Research RNA extraction kit. Briefly, embryos underwent freeze-thaw lysis followed by RNA isolation following the manufacturer’s protocol. Complementary DNA synthesis was performed according to manufacturer’s protocol (High-Capacity cDNA Reverse Transcription Kit, ThermoFisher). cDNA was subsequently amplified by qRT-PCR using gene-specific TAQMan probes: *CASP3*, *CASP7*, *CASP8*, *BCL2A1*, *NFKBIA*, *NFKBIZ, GAPDH*, *ACTB, 18S* (ThermoFisher). All reactions were performed in duplicate to ensure reproducibility. Cycle threshold (C_t_) values were recorded for each target gene following established protocols (Lal et al., 2017).

### Data processing

The C_t_ values for apoptosis-related genes were normalized to a housekeeping gene (*18S*) with stable expression across all experimental conditions to generate ΔC_t_ values. Fold changes in gene expression were calculated either using the log 2^(−ΔCt)^ for untreated samples or log 2^(−ΔΔCt)^ when PAF-treated and untreated samples are compared. For each set of samples, two biological replicates were used and each biological sample was run in duplicate. Microsoft Excel was used to calculate the Standard Error of the Mean (SEM) for each gene with or without PAF. ANOVA was performed using SigmaStat for Windows (version 4.0, 2016).

## Results

### Housekeeping gene selection

**FIGURE 2:**
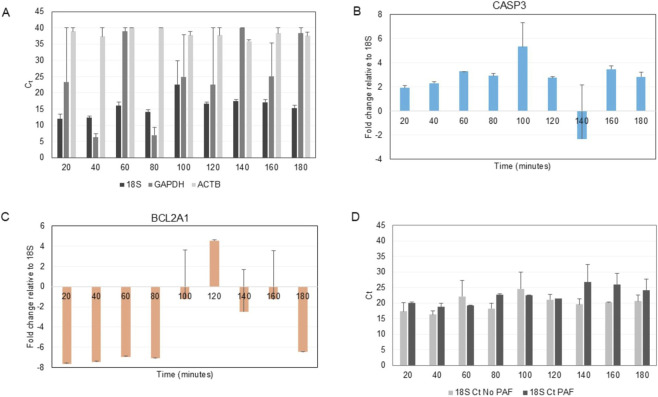
Relative gene expression for preliminary experiments. **(A)** Housekeeping genes (*18S*, *GAPDH* and *ACTB*) show steady expression of *18S* compared to fluctuating *GAPDH* and low expression of *ACTB*. C_t_ values of *18S* (black bars), *GAPDH* (gray bars), and *ACTB* (white bars) measured across time points from 20 to 180 min. A one-way ANOVA of *18S* expression across time points 20–180 min shows no significant difference across time points: F (11, 12) = 0.774; p = 0.661. **(B)** *CASP3* relative gene expression over time reported as fold change compared to *18S*. A one-way ANOVA was used to examine the effect of time on *CASP3* expression and showed no significant effect of time on expression: (F11,12) = 0.774, p = 0.661. **(C)** *BCL2A1* relative gene expression over time reported as fold change compared to *18S*. A one-way ANOVA of *BCL2A1* expression showed a significant effect of time on expression: (F11,12) = 3.03, p = 0.0345. A Tukey HSD post-hoc comparison revealed three pairwise comparisons were statistically significant: 80 versus 100 min (p = 0.030), 80 versus 180 min (p = 0.048) and 100 versus 240 min (p = 0.0347). **(D)** *18S* relative gene expression with and without PAF. C_t_ values of *18S* in PAF-treated cells (black bars) versus untreated controls (gray bars) across time points from 20 to 180 min. A two-way ANOVA was used to examine the effect of time and presence of PAF on *18S* expression*.* The ANOVA revealed that there was a difference in 18S expression between the PAF and no PAF groups, however, the shape of the response over time was statistically similar between the PAF and untreated groups (p = 0.225).

To ensure accurate quantification of gene expression changes, we evaluated the stability of three candidate housekeeping genes: *GAPDH*, *ACTB*, and *18S* (Figure 2A). *GAPDH* exhibited substantial fluctuation in C_t_ values across the time course (3 h post fertilization), indicating variable expression that would introduce normalization errors. *ACTB* demonstrated consistently high C_t_ values (>30 cycles), suggesting its expression levels were too low for reliable detection. In contrast, *18S* maintained stable, moderate C_t_ values (approximately 18–22 cycles) throughout the experimental time course, with minimal variation between time points; therefore, *18S* was selected as the suitable gene for normalization. A one-way ANOVA of *18S* expression across time points 20–180 min shows no significant difference across time points: F (11, 12) = 0.774; p = 0.661. Since p > 0.05, expression of 18S does not significantly change across time points.

Preliminary analysis of apoptotic gene expression using fertilized embryos from *L. variegatus* revealed distinctly different expression patterns for apoptosis-promoting and apoptosis-inhibiting genes (Figures 2B,C). Apoptosis-promoting gene, *CASP3*, exhibited elevated expression levels compared to *18S* for up to 3 h. A one-way ANOVA was used to examine the effect of time on *CASP3* expression and showed no significant effect of time on expression: (F11,12) = 0.774, p = 0.661. Conversely, the apoptosis-inhibiting gene, *BCL2A1*, displayed reduced basal expression compared to *18S*. A one-way ANOVA was used to examine the effect of time on *BCL2A1* expression and showed a significant effect of time on expression: (F11,12) = 3.03, p = 0.0345. A Tukey HSD post-hoc comparison revealed three pairwise comparisons were statistically significant: 80 versus 100 min (p = 0.030), 80 versus 180 min (p = 0.048) and 100 versus 240 min (p = 0.0347). This baseline expression pattern established that apoptotic machinery is transcriptionally active during early sea urchin embryogenesis, with the balance favoring pro-apoptotic signaling under control conditions (without PAF treatment).

### PAF does not affect housekeeping gene expression

Next, we investigated the effect of PAF treatment on both apoptosis-promoting and inhibiting genes. To validate that PAF treatment did not introduce systematic artifacts into our gene expression measurements, we compared *18S* expression between PAF-treated and control embryos. The C_t_ values for *18S* remained stable in both experimental groups, with no significant deviation between PAF-exposed and untreated embryos at any time point examined (Figure 2D). A two-way ANOVA was used to examine the effect of time and presence of PAF on *18S* expression*.* The ANOVA revealed that there was a difference in 18S expression between the PAF and no PAF groups, however, the shape of the response over time was statistically similar between the PAF and untreated groups (p = 0.225). Therefore PAF acts as an additive effect for *18S* expression, but is not time-dependent.

### PAF increases expression of apoptosis-promoting genes

**FIGURE 3:**
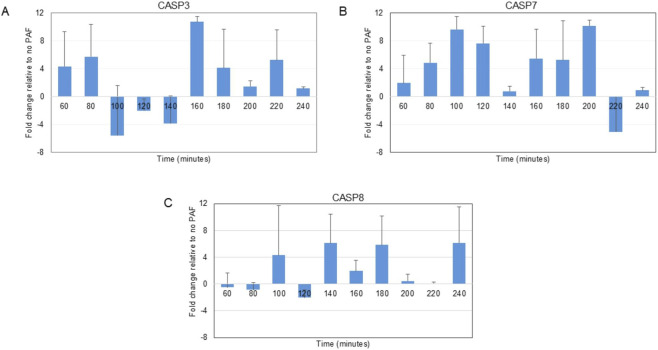
Relative expression of apoptosis-promoting genes in PAF-treated versus untreated cells. Fold change over time for **(A)** *CASP3*, **(B)** *CASP7*, and **(C)** *CASP8*. Error bars represent standard error of the mean. A two-way ANOVA showed no significant effect of PAF and/or time on *CASP3* expression (PAF, p = 0.148; time, p = 0.533; PAF x time, p = 0.356). A two-way ANOVA revealed a significant effect of PAF on *CASP7* expression across all time points: F = 9.89, p = 0.0051. There was no statistically significant effect of time (p = 0.288) on *CASP7* expression. A two-way ANOVA showed no significant effect of PAF on *CASP8* expression across all time points: p = 0.104. There was no statistically significant effect of time (p = 0.727) on *CASP8* expression.

To address whether PAF treatment changes the expression pattern of apoptosis-promoting and apoptosis-inhibiting genes, *CASP3, CASP7* and *CASP8* (Ai et al., 2024), RNA was collected from PAF-treated and control urchin embryos in 20-min increments after PAF treatment for up to 3 hours. Fold change was calculated to compare PAF-treated embryos to controls (no PAF) and revealed that PAF exposure significantly increased expression of apoptosis-promoting genes during early development (Figure 3). All three caspase genes examined demonstrated predominantly positive expression changes, indicating upregulation in response to PAF treatment. *Caspase-3* (*CASP3*) exhibited substantial variability following a sinusoidal pattern across the time course, with an elevated level at 60 min followed by some downregulation, and upregulation at 160–240 min. A two-way ANOVA showed no significant effect of PAF and/or time on *CASP3* expression (PAF, p = 0.148; time, p = 0.533; PAF x time, p = 0.356). *Caspase-7* (*CASP7*) displayed the most robust and consistent upregulation pattern, with positive expression changes at nearly all time points examined, achieving maximum expression at 200 min. However, a two-way ANOVA revealed a significant effect of PAF on *CASP7* expression across all time points: F = 9.89, p = 0.0051. There was no statistically significant effect of time (p = 0.288) on *CASP7* expression. *Caspase-8* (*CASP8*) showed a more moderate upregulation pattern between 120–180 min, with some variability during other time points. A two-way ANOVA showed no significant effect of PAF on *CASP8* expression across all time points: p = 0.104. There was no statistically significant effect of time (p = 0.727) on *CASP8* expression. The overall pattern demonstrates that PAF treatment paradoxically enhances transcription of pro-apoptotic executioner and initiator caspases, contrary to expectations based on PAF’s documented role as a survival factor in mammalian systems.

### PAF exerts biphasic effects on apoptosis-inhibiting genes

**FIGURE 4:**
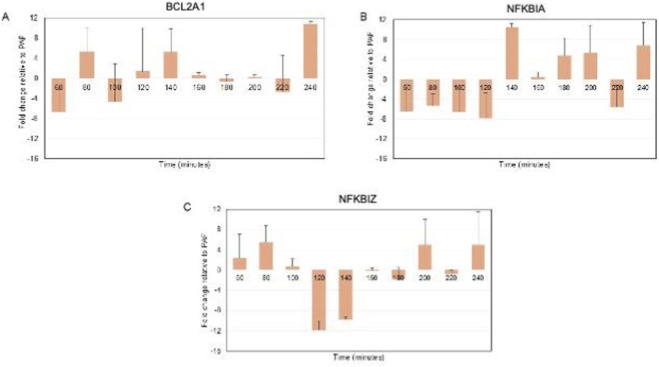
Relative expression of apoptosis-inhibiting genes in PAF-treated versus untreated cells. Fold change over time for, **(A)** *BCL2A1*, **(B)** *NFKBIA*, and **(C)** *NFKBIZ*. Error bars represent standard error of the mean. A two-way ANOVA showed no significant effect of PAF on *BCL2A1* expression across all time points: p = 0.477. There was a very close to significant effect of time on *BCL2A1* expression, where p = 0.0525. A two-way ANOVA showed no significant effect of PAF on *NFKBIA* expression across all time points: p = 0.784. There was no statistically significant effect of time (p = 0.973) on *NFKBIA* expression. A two-way ANOVA showed no significant effect of PAF on *NFKBIZ* expression across all time points: p = 0.699. There was no statistically significant effect of time (p = 0.525) on *NFKBIZ* expression.

Analysis of apoptosis-inhibiting genes *BCL2A1, NFKBIA* and *NFKBIZ* (Ai et al., 2024; Vogler, 2012; Galasso et al., 2019) revealed complex, gene-specific responses to PAF treatment (Figure 4). *BCL2A1*, an anti-apoptotic member of the BCL-2 family, demonstrated the most variable response pattern throughout the time course of the experiment. A two-way ANOVA showed no significant effect of PAF on *BCL2A1* expression across all time points: p = 0.477. However, there was a very close to significant effect of time on *BCL2A1* expression, where p = 0.0525. *NFKBIA*, encoding transcription factor critical for cell survival signaling, showed sustained suppression during the first hour of PAF treatment, with recovery beginning around 140 min. A two-way ANOVA showed no significant effect of PAF on *NFKBIA* expression across all time points: p = 0.784. There was no statistically significant effect of time (p = 0.973) on *NFKBIA* expression. However, there was a close to significant effect of PAF x Time where p = 0.053. *NFKBIZ* displayed the major variation throughout the time course, with significant suppression between 100–140 min, followed by an upregulation at later time points. A two-way ANOVA showed no significant effect of PAF on *NFKBIZ* expression across all time points: p = 0.699. There was no statistically significant effect of time (p = 0.525) on *NFKBIZ* expression. Collectively, these data indicate that PAF initially suppresses anti-apoptotic and cell survival signaling pathways before triggering compensatory mechanisms that restore or enhance expression of these protective genes as embryonic development progresses beyond 140 min post-fertilization.

## Discussion

Our results reveal a more complex regulatory mechanism than initially hypothesized, demonstrating that PAF orchestrates selective effects on pro- and anti-apoptotic genes during early sea urchin embryo development. Among apoptosis-promoting genes, *CASP7* showed statistically significant upregulation in response to PAF treatment (p = 0.0051), while *CASP3* and *CASP8* exhibited non-significant trends. For apoptosis-inhibiting genes, *BCL2A1* displayed significant time-dependent expression changes (p = 0.0345 in controls; p = 0.0525 with PAF), while *NFKBIA* and *NFKBIZ* showed variable patterns without statistical significance. Critically, where temporal changes occurred, both control and PAF-treated embryos exhibited similarly shaped expression curves with changes at approximately the same developmental time points, indicating that PAF modulates expression amplitude while preserving normal developmental timing.

Previous studies have demonstrated that sea urchin oocytes, eggs, and early embryos possess functional apoptotic machinery capable of activation under experimental conditions, establishing that the molecular components for programmed cell death are present from the earliest developmental stages (Voronina and Wessel, 2001). PAF’s sustained upregulation of *CASP7*, combined with *BCL2A1*’s maintenance of its time-dependent expression pattern (though with modulated amplitude in the presence of PAF), suggests that PAF enhances apoptotic capacity for quality control without disrupting the intrinsic developmental program. The parallel expression trajectories in control and treated embryos demonstrate that PAF does not alter developmental timing but rather amplifies specific apoptotic machinery components within the normal temporal framework.

The preservation of temporal patterns has important mechanistic implications. It suggests that PAF does not fundamentally alter transcriptional regulatory networks controlling developmental timing but rather adjusts expression levels of specific genes—significantly elevating *CASP7* while maintaining *BCL2A1*’s developmental stage-appropriate fluctuations. This creates a regulatory landscape where apoptotic machinery is transcriptionally enhanced but remains subject to normal developmental controls. The similar temporal curves between treatment groups indicate that upstream signals regulating gene expression timing (such as cell cycle progression or maternal-to-zygotic transition events) remain intact in PAF-treated embryos.

These results help reconcile our observations with Lal et al.'s (2017) report of reduced Caspase-3 activity at 24 h in PAF-treated embryos. While *CASP7* expression is elevated during early development (0–4 h), the preserved temporal dynamics of *BCL2A1* mean that anti-apoptotic protection continues increasing at later stages according to the normal developmental program. By 24 h, this naturally increasing anti-apoptotic protection could functionally inhibit caspase activity despite earlier transcriptional enhancement. Similarly, our data mechanistically support that rigorous early quality control ensures only viable cells proceed through subsequent stages, allowing accelerated development of healthy mammalian embryos (Roudebush et al., 2002; 2003). PAF acts beyond a simple “survival factor” (O'Neill, 2005; O'Neill, 2008) but as a selective developmental modulator that enhances quality control capacity during critical windows while maintaining normal temporal progression.

The temporal dynamics observed have significant implications for assisted reproductive technologies. The finding that *BCL2A1* and other apoptosis inhibiting genes follow similar trajectories regardless of PAF treatment suggests optimal developmental windows for quality control are intrinsically programmed. PAF supplementation enhances quality control efficiency during these windows by elevating *CASP7* without disrupting protective mechanism timing. Understanding these expression patterns—both amplitude changes and preserved temporal dynamics—could enable clinicians to make informed decisions about embryo selection and supplementation timing, potentially improving *In Vitro* Fertilization (IVF) success rates by ensuring both adequate quality control and developmental progression.

## Conclusion

This study reveals that PAF regulates early embryonic development through selective amplitude modulation: significantly upregulating *CASP7* (p = 0.0051) while preserving the natural time-dependent expression patterns of *BCL2A1* (p = 0.0345 in controls; p = 0.0525 with PAF). Where temporal changes occurred, control and PAF-treated embryos exhibited similarly shaped curves with changes at approximately the same developmental time points. This demonstrates that PAF enhances specific pro-apoptotic components without disrupting intrinsic temporal regulatory programs governing normal embryogenesis. These findings provide a mechanistic explanation for PAF’s role in producing healthier, more rapidly developing embryos and suggest that PAF supplementation could improve assisted reproductive technology outcomes by enhancing quality control capacity within the existing developmental framework.

While we used both technical and biological duplicate measurements for all qRT-PCR assays, additional biological replicates would strengthen statistical power and better characterize temporal patterns. Our population-based measurements may mask individual variation in developmental timing and gene expression responses. Additionally, our study focused exclusively on gene expression at the transcriptional level without assessing protein abundance or enzymatic activity at these early time points.

Future studies could extend this time-course analysis beyond 4 h through gastrulation to determine whether preserved temporal patterns persist and directly connect our early time points with Lal et al.'s (2017) measurements at 24 h. Measuring protein levels and enzymatic activity—particularly caspase-7 activity and BCL2A1 protein abundance—would establish whether transcriptional patterns translate to functional changes. Investigating how environmental stressors such as temperature fluctuations and pH changes modify both PAF’s amplitude effects and temporal patterns would provide insights into regulatory mechanism robustness.

Most importantly, translating these findings to IVF embryos is a possibility as gene expression can be measured in spent culture media. This could allow researchers to test whether mammalian embryos show similar temporal patterns and whether PAF elevates specific pro-apoptotic genes while preserving normal developmental timing in early human embryo development. If these patterns are observed in human embryos, then expression profiles showing both appropriate temporal coordination and optimal amplitude of apoptotic gene expression could 1 day help clinicians select the most viable embryos for uterine transfer. The finding that PAF modulates amplitude while preserving timing suggests continuous supplementation during early culture may be beneficial. Additionally, the apoptotic gene expression profile in embryos could potentially be optimized with PAF supplementation during embryo culture, though extensive validation demonstrating correlation with developmental competence and pregnancy outcomes would be essential.

## Data Availability

Datasets are available on request: The raw data supporting the conclusions of this article will be made available by the authors, without undue reservation.
